# African swine fever control and prevention: an update on vaccine development

**DOI:** 10.1080/22221751.2022.2108342

**Published:** 2022-08-25

**Authors:** Ana Catarina Urbano, Fernando Ferreira

**Affiliations:** a CIISA – Centre for Interdisciplinary Research in Animal Health, Faculty of Veterinary Medicine, University of Lisbon, Lisboa, Portugal; b Associate Laboratory for Animal and Veterinary Sciences (AL4AnimalS), Lisboa, Portugal

**Keywords:** African swine fever, control, domestic pigs, immune response, live attenuated, vaccine, review, wild boar

## Abstract

African swine fever (ASF) is a lethal and highly contagious viral disease of domestic and wild pigs, listed as a notifiable disease reported to the World Organization for Animal Health (OIE). Despite its limited host range and absent zoonotic potential, the socio-economic and environmental impact of ASF is very high, representing a serious threat to the global swine industry and the many stakeholders involved. Currently, only control and eradication measures based mainly on early detection and strict stamping-out policies are available, however, the rapid spread of the disease in new countries, and in new regions in countries already affected, show these strategies to be lacking. In this review, we discuss approaches to ASF vaccinology, with emphasis on the advances made over the last decade, including the development of virulence-associated gene deleted strains such as the very promising ASFV-G-ΔI177L/ΔLVR, that replicates efficiently in a stable porcine epithelial cell line, and the cross-protecting BA71ΔCD2 capable of stably growing in the commercial COS-1 cell line, or the naturally attenuated Lv17/WB/Rie1 which shows solid protection in wild boar. We also consider the key constraints involved in the scale-up and commercialization of promising live attenuated and virus-vectored vaccine candidates, namely cross-protection, safety, lack of suitable animal models, compatibility with wildlife immunization, availability of established and licensed cell lines, and differentiating infected from vaccinated animals (DIVA) strategy.

## GRAPHICAL ABSTRACT

**Figure:**
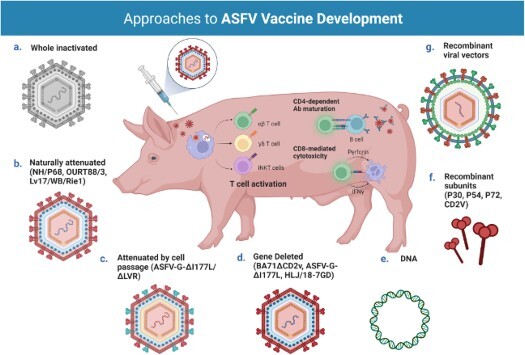

## Introduction

African swine fever (ASF) is a highly infectious and severe haemorrhagic viral disease of pigs, endemic to South-Saharan Africa (24 genotypes based on the sequence of the c-terminus of the p72 surface antigen) and the island of Sardinia in Italy (p72 genotype I). The transcontinental spread of ASF occurred on at least three separate occasions, most significantly to Georgia, in 2007, where it spread from the Black Sea port of Poti across the Caucasus region into the Russian Federation (RF) and Eastern Europe [1]. The following decade saw the disease become epizootic in the RF, and by 2018 it had spread as far west as Belgium and east to the People’s Republic of China, quickly taking over most of Southeast Asia and Oceania [2,3]. Since then, the epidemiological situation of ASF has continued to deteriorate; in July 2021, after an absence of nearly 40 years, ASF was introduced in the Dominican Republic, and later Haiti, and in January 2022 reappeared on the Italian mainland. Several reoccurrences have since been reported in China, the RF, Moldova, and Ukraine, and North Macedonia reported their first occurrence, as did Thailand, one of the few countries in the region that had remained unscathed [3]. These recent events highlight an extremely disconcerting pattern of continuous spread, exacerbated by the fact that in many of these regions small-scale and semi-industrial farms account for the majority of pig production. As such, outbreaks carry severe socio-economic consequences, causing devastation of rural livelihoods dependent on livestock production, and threatening overall market stability and food security.

The African Swine Fever Virus (ASFV) is a large, double-stranded nucleocytoplasmic DNA arbovirus, the only member of the *Asfarviridae* family. Virions have a diameter of around 250 nm and consist of a central nucleoid enclosed by an icosahedral protein capsid (or core shell), an internal lipoprotein membrane (or inner envelope), an icosahedral protein outer capsid, and a dispensable external lipoprotein envelope (or outer envelope) that is obtained when the virus buds out through the plasma membrane [4]. Both intracellular and extracellular viral forms are infectious. Its natural host range is limited to soft-bodied ticks of the genus *Ornithodoros* and members of the family *Suidae*, where it replicates mainly in cells of the mononuclear phagocytic system, resident macrophages, and specific reticular cells. Significantly, African wild pigs are not affected. In domestic pigs and wild boar, however, clinical signs vary considerably, and the individual outcome can range from fatal to subclinical. Depending on the virulence of the strain involved, a graded series of forms occurs, with lethality ranging from 100% (peracute form) to <30% (chronic form). The genotype II strain currently affecting Europe and Asia is highly virulent, causing the acute form of the disease, although there is evidence that some reduced-virulence isolates may be circulating among wild boar in the Baltic States and domestic pigs in China, with reports of both naturally mutated genotype II low virulent strains and genotype I low virulent epidemic strains detected in the field [5,6]. The latter have longer incubation periods, cause chronic disease, and infected pigs shed virus continuously, making early diagnosis more difficult. We refer the reader to several recent reviews that detail the epidemiology and control of ASFV infection [7–11].

## Immune response against ASF

Protective immunity against ASFV is poorly understood. As with most viral infections, innate immunity and both humoral and cellular responses appear to be important for protection. [NO_PRINTED_FORM][NO_PRINTED_FORM]Despite original reports indicating a lack of neutralizing activity of antibodies against ASFV, evidence of neutralizing antibodies against the virus seem to be overwhelming [7,12]. Antibody-mediated neutralization has some uncommon characteristics, however, including loss of susceptibility to neutralization by cell culture passage because of changes in the phospholipid composition of viral membranes and/or the presence of sera blocking antibodies that inhibit complete neutralization [12]. The ASFV hemagglutinin CD2v/EP402R is perhaps the viral protein most significantly implicated in protective immunity [11,13]. CD2v, together with the auxiliary HA viral antigen, C-type Lectin/EP153R, is also among the most variable gene orthologues between ASFV isolates, providing potentially significant antigens in serogroup-specific immunity. ASFV hemadsorption inhibiting (HAI) serotype-specific protective immunity is a recent concept supported by data indicating that p72-genotyping does not fully correlate with either homologous or heterologous cross-protection, as distinct strains are sometimes able to induce measures of cross-protection, while strains that appear closely related fail to cross-protect. Recent findings demonstrate that CD2v and C-type lectin are necessary and sufficient for mediating HAI serologic specificity and that CD2v/C-type lectin genotyping can reliably group ASFV strains by serogroup [14]. While important for mediating cross-protective responses *in vivo*, however, they do not confer complete serotype-specific homologous protection [14,15], and several studies show that attenuated ASFV viruses containing CD2 gene deletion or N-terminal truncating mutations in CD2v/C-type lectin genes (thus, presumably, lacking the proteins) protect pigs and wild boar from virulent virus challenge [16–18], indicating that additional protective antigens need to be identified. Other potential viral neutralizing epitopes include viral capsid proteins p30/CP204L, p54/E183L, and p72/B646L, viral proteins B602L, C44L, CP312R, E183Lp, K145R, and K205R, as well as the structural proteins A104R, p10/K78R, and the non-structural proteins ribonucleotide reductase (F334L, F778Rp), DNA ligase (NP419L), and thymidine kinase (TK/K169R) [11]. In addition to antibody-mediated virus neutralization, other potentially protective roles of ASFV antibodies have been indicated by mechanisms such as antibody-dependent cell-mediated cytotoxicity (ADCC) and complement-mediated cytotoxicity (CDC) [11,12,19,20].

Cellular immunity is also likely to be essential for protection. Key roles have been indicated for natural killer (NK) and CD8^+^ T cells; Leitão et al. [18] demonstrated elevated NK cell activity in pigs infected with the naturally attenuated NHV/NHP68 strain, and that the animals subsequently survived challenge with the highly virulent L60 isolate, while Alonso et al. [21] showed CD8-dependent lysis of ASFV-infected cells in experimental models with attenuated virus isolates and Oura et al. [16] that antibody-mediated depletion of this cell subset abrogated protection. Schäfer et al. [22] and Hür et al. [23] additionally demonstrated that the immune response in wild boar and domestic pigs is based primarily on increases of CD4^−^/CD8α^+^ during infection with moderately virulent ASFV and of CD4^+^/ CD8α^+^ (DP) T cells during infection with the highly virulent Armenia08 isolate. Further, increases in effector γδ T cell frequencies in wild boar were suggested as an explanation for the higher virulence in this subspecies. The authors also demonstrated significant alterations in invariant NK T cell (iNKT) frequency [24] and activation patterns [22] during infection, supporting the findings of Leitão et al. and the notion that this cell subset takes part in the antiviral response against ASFV. Despite complications arising from the heterogeneity of the T cell population [25] and the variability of MHC peptide presentation within the outbred pig population [11], ASFV-specific CD8^+^ T cell determinants with protective potential have been identified in the G1340L, p30, p72, CD2v, and C-type lectin viral proteins [15,21,26–29]; their role in protection is not fully elucidated, however. Further work towards the identification of other CD8^+^ T cell determinants in both attenuated and virulent strains and clarification of their protective potential is being developed [30,31]. In conclusion, the evidence available indicates that induction of protection involves both antibody- and cell-mediated mechanisms, but the antigens and types of cellular responses required need further characterization. For more detailed information on cellular and humoral immune responses to ASFV infection we direct readers to two excellent reviews [12,32].

## ASF vaccine approaches

### Inactivated vaccines

**Figure 1.**
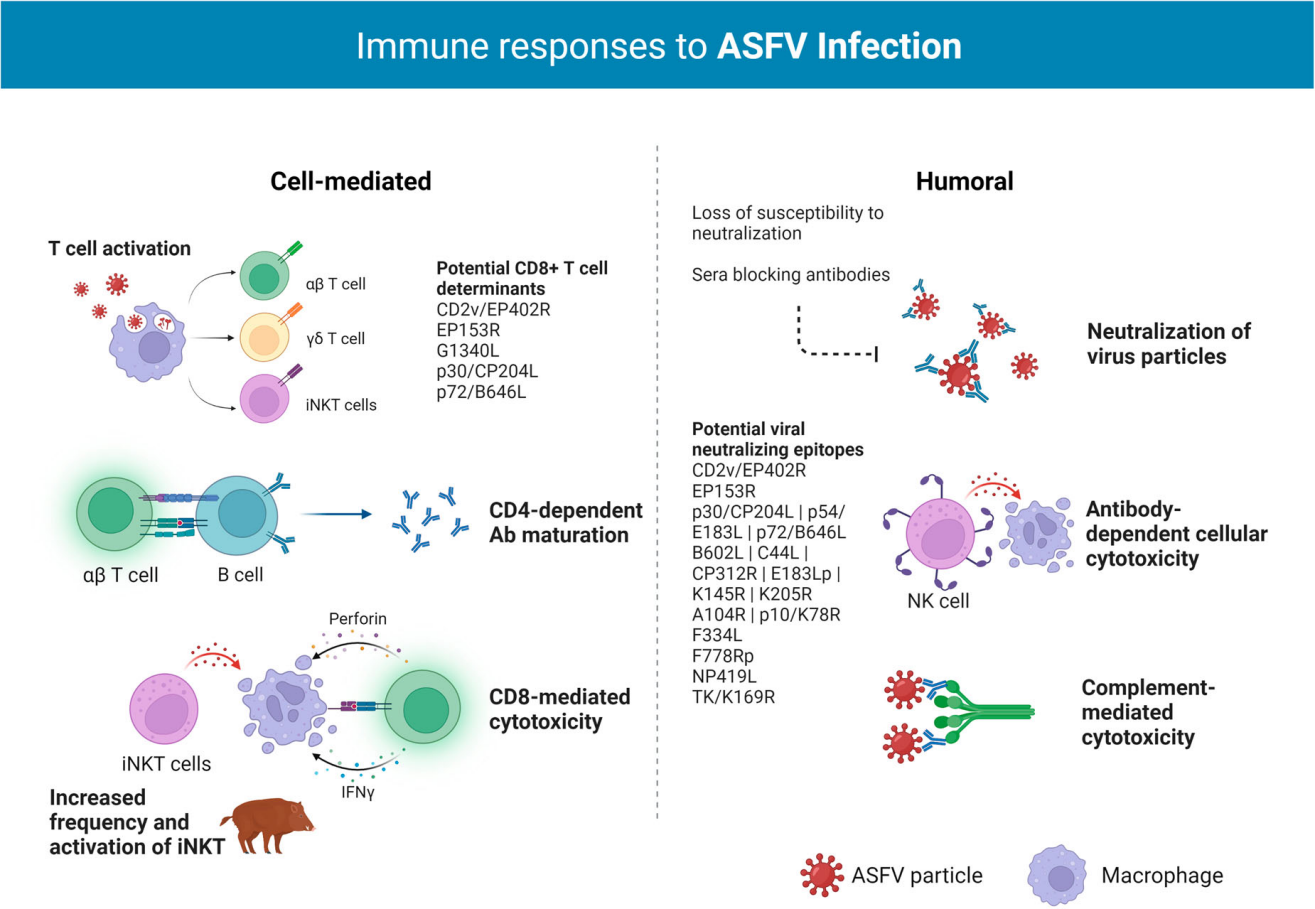
Immune responses to ASFV infection. Both humoral and cellular immune responses appear to be important for protection against ASFV infection. T cells have been shown to play a particularly important role in survival with key roles identified for natural killer (NK) and CD8+ T cells. CD4+ T helper cells seem to support B cell responses and essential antibody (Ab) maturation, particularly in infection with highly virulent isolates. Studies on nonconventional T cells, such as effector γδ T cells and invariant Natural Killer T (iNKT) cells, indicate these cell subsets also take part in the antiviral response against ASFV. In wild boar, the significant bias towards γδ T cells has been suggested as an explanation for the higher disease severity and lethality in this species. Several studies have also revealed the relevance of antibodies in the protection against ASF. Antibody-mediated neutralization has some uncommon characteristics in ASFV infection, namely loss of susceptibility to neutralization by cell culture passage because of changes in the phospholipid composition of viral membranes and/or the presence of sera blocking antibodies that inhibit complete neutralization. A number of ASFV proteins have been implicated in the induction of neutralizing antibodies during infection, most notably the ASFV hemagglutinin CD2v/EP402R. Other antibody driven protective mechanisms include antibody-dependent cell-mediated cytotoxicity (ADCC) and complement-mediated cytotoxicity (CDC). Created with BioRender.com (accessed on 01 July 2022).

**Figure 2.**
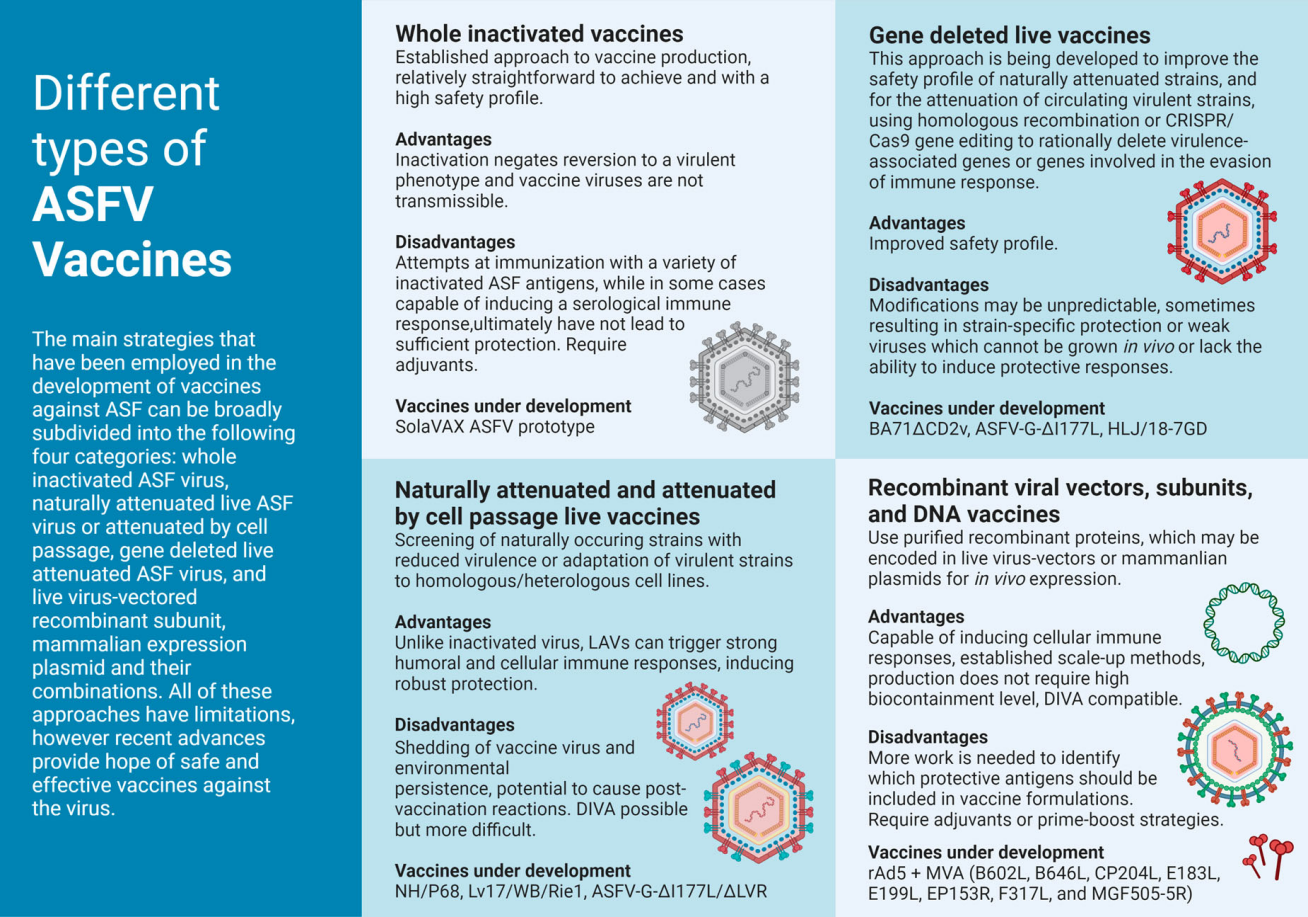
Different approaches for the development of vaccine candidates against ASFV. Three main strategies have been employed in the development of ASFV vaccine candidates: whole inactivated ASF virus vaccines; live virus-vectored recombinant, subunit, and mammalian expression plasmid vaccines; and live attenuated virus vaccines (LAVs), which we have further subcategorized into naturally attenuated or attenuated by cell passage, and gene deleted vaccines. The figure highlights the main advantages and disadvantages of each approach, as well as existing examples under development. DIVA – Differentiating Infected from Vaccinated Animals. Created with BioRender.com (accessed on 01 July 2022).

Virus inactivation is an established approach to vaccine production, relatively straightforward to achieve and with a higher safety profile when compared to live vaccines. The inactivation process negates reversion to a virulent phenotype and vaccine viruses are nontransmissible, the two major drawbacks of attenuated vaccines. Inactivation, however, does not necessarily translate into a vaccine that elicits protective immunity. Attempts at immunization of pigs with a variety of inactivated ASF antigens, using traditional methods, while in some cases capable of inducing a serological immune response, ultimately did not lead to sufficient protection [33,34]. This is perhaps not entirely surprising, given that cellular immunity seems essential for protection and that effective virus neutralization is difficult to achieve in primary infections [11]. Researchers at the International Livestock Research Institute in Kenya (ILRI) in collaboration with Colorado State University (CSU) are now attempting to develop an inactivated vaccine using a novel method [35], originally developed for the treatment of blood products. If successful, benefits will include a well-established toxicological safety profile, with little to no toxicity or disposal risk to facility personnel or the environment, and the rapid and affordable production of vaccine candidates using commercially available equipment, reagents, and disposables, which makes it attainable in both high- and low- income locations around the world, as close to endemic areas as possible. A variety of ASFV vaccine preparations, using specific adjuvants, are currently being assessed for immune responses Figures 1 and 2.

### Subunit, DNA and virus-vectored vaccines

Subunit vaccines use purified recombinant proteins or synthetic peptides encoding specific viral epitopes capable of inducing a protective immune response. This is accomplished by using conventional biochemical or recombinant DNA technologies to generate an antigen that is formulated with an adjuvant. Alternatively, the technology can be used to generate a plasmid or a live virus-vectored recombinant construct containing the DNA encoding the antigen(s) of interest for *in vivo* expression. These DNA and viral-vectored vaccines have an advantage over antigen-based inactivated formulations in that they are capable of inducing cell-mediated immune responses. Moreover, viral vectors have the added advantage of being able to actively penetrate the host’s cells and replicate as a live attenuated vaccine (LAV) and are well-suited for differentiating infected from vaccinated animals (DIVA) with vector encoded immunogens serving as vaccine markers Tables 1 and 2.

**Table 1.** Summary of the number of outbreaks and animal losses caused by ASF in the different world regions (2016-2022). Data reported since January 2020 covers only epizootic situation. * Losses (deaths + animals killed and disposed of): this figure refers to losses in the establishments affected by the outbreaks and it does not include the animals culled in areas around the outbreak for controlling the disease. Source WHOA/OIE WAHIS.

	Outbreaks		Cases		Losses*	Total Outbreaks
	Domestic pigs	Wild boar	Domestic pigs	Wild boar	Domestic pigs	
Africa	277		74 085		105 509	277
Americas	210		8 592		14 972	210
Asia	10 967	2 149	204 344	2 746	7 132 038	13 116
Europe	7 607	33 565	1 553 645	57 185	2 643 923	41 172
Oceania	4		500		397	4
Total	19 065	35 714	1 841 166	59 931	9 896 839	54 779

**Table 2.** Promising Live Attenuated Vaccines developed in 2015–2022. PBMs porcine blood monocyte/macrophages; BMs pig bone marrow cells; COS-1 monkey kidney tissue-derived cells; PAMs primary porcine alveolar macrophages; PIPEC Plum Island porcine epithelial cells, a porcine fetal kidney cell line engineered to express the bovine αVβ6 integrin.

Candidate	ASFV strain	Virulence	p72 genotype	Attenuation strategy	Protection	Production system	References
NH/P68	NH/P68	High	I	Naturally attenuated	Homologous and heterologous strain (L60, Arm07)	PBMs	Gallardo et al., 2012; Leitão et al., 2001a
OURT88/3	OURT88/3	Low	I	Naturally attenuated	Homologous and heterologous strain (OURT88/1, Ug65)	BMs	Boinas et al., 2004b; King et al., 2011; Mulumba-Mfumu et al., 2016; Sánchez-Cordón et al., 2017
Lv17/WB/Rie1	Lv17/WB/Rie1	Low	II	Naturally attenuated	Homologous strain (Armo7)	PBMs	Barasona et al., 2019; Gallardo et al., 2019
BA71ΔCD2v	BA71	Low	I	Gene deleted (CD2v)	Homologous and heterologous strain (E75, Georgia 2007)	COS-1	Lopez et al., 2020; Monteagudo et al., 2017
HLJ/18-7GD	HLJ/18	High	II	Gene deleted (MGF505-1R, MGF360-12L, MGF360-13L, MGF360-14L, MGF505-2R, MGF505-3R, and CD2v)	Homologous strain (ASFV HLJ/18)	PAMs	Chen et al., 2020
ASFV-G-ΔI177L	Georgia 2007	High	II	Gene deleted (I177L)	Homologous strain (Georgia 2007)	PAMs	Borca, Ramirez-Medina, et al., 2020
ASFV-G-ΔI177L/ΔLVR	ASFV-G-ΔI177L	High	II	Gene deleted (I177L) and cell passage	Homologous strain (Georgia 2007)	PIPEC	Borca, Rai, et al., 2021
SY18ΔI226R	ASFV-SY18	High	II	Gene deleted (I226R)	Homologous strain (ASFV-SY18)	PAMs	Zhang et al., 2021
ASFV-G-ΔA137R	Georgia 2010	High	II	Gene deleted (A137R)	Homologous strain (Georgia 2010)	PAMs	Gladue et al., 2021
ASFV-G-ΔE184L	Georgia 2010	High	II	Gene deleted (E184L)	Homologous strain (Georgia 2010)	PAMs	Ramirez-Medina et al., 2022

As described in the previous section, several ASFV antigens have been evaluated for their protective potential, including structural proteins p54, p30, p72 and the hemagglutinin CD2v, and these proteins have traditionally been the main targets of subunit and DNA vaccine strategies. Preliminary antigen-based vaccination experiments using recombinant p54 and p30 expressed in baculovirus conferred a variable degree of protection against lethal challenge, ranging from a delay in disease onset to complete protection [36]. A chimeric p54/p30 expressed in the same system also had some success, with pigs developing neutralizing antibodies and surviving challenge with the virulent virus [37]. In a different study, however, a combination of baculovirus-expressed p54, p30, and p72 failed to induce protection against challenge [38]. Baculovirus-expressed CD2v also demonstrated some degree of protection against challenge with the virulent virus [13], though protection was induced in the absence of neutralizing antibodies.

Contrary to what was shown with the antigen-based subunit formulations recent work with a DNA vaccine encoding a p54/p30 fusion protein failed to induce protection, producing neither neutralizing antibodies nor T cell responses [29] and while specific T cells against ASFV proteins were detected in pigs vaccinated with a construct encoding a fusion of the swine leukocyte antigen II (SLA-II) with p54/p30, they were also not protected from challenge [39]. Fusion of a gene fragment encoding the extracellular soluble domain of CD2v with the p54/p30 chimaera, to replicate the protection previously observed, induced both humoral and cellular responses in pigs but was ultimately not successful; however, a fusion of ubiquitin to the three ASFV determinants induced a strong CD8^+^ T cell response and conferred partial protection in the absence of specific antibodies [29]. Immunization of pigs with an ASFV expression library containing over 4000 random DNA fragments fused to ubiquitin, excluding the p54, p30, and CD2v open reading frames, also conferred partial protection [40]. In this case, protection likewise correlated with the detection of specific T cell responses, indicating that these antigens, though necessary, are likely not sufficient for protection.

Still more recent studies with antigen cocktails of up to 47 different ASFV genes delivered by adenovirus, alphavirus and vaccinia virus vectors were able to demonstrate induction of strong antigen-specific cellular responses [31,41–43]. One study reported that a pool comprised of eight ASFV genes including B646L (p72), CP204L (p30), CP530R (pp62), and MGFs 110-4L and 110–5 vectored by replication-deficient human adenovirus 5 prime and modified vaccinia Ankara boost led to reduced clinical signs and reduced levels of viremia in a proportion of pigs after challenge with the virulent OUR T88/1 isolate [31]. A combination of the ASFV genes B646L (p72), CP204L (p30), CP530R (pp62), and E183L (p54) with the mature p37 product and two sections of the mature p150 protein of the pp220 polyprotein (CP2475L gene) using the same prime-boost strategy with two different adjuvants were evaluated in another study [41], with roughly half of the animals in one of the adjuvanted groups surviving challenge with the Georgia 2007/1 isolate. A follow-up study [30] of two immunization protocols using pools of eight of these viral vectored ASFV genes ultimately achieved 100% protection from fatal disease in animals challenged with the OUR T88/1 strain.

In the long run, subunit, DNA, and viral vectored vaccines show promise and several candidates have been shown to induce specific humoral and/or cellular immune responses which appear to confer partial to full protection. However, the different nature of the immunization protocols used in these studies, including the type of vaccine, vaccination strategy and challenge model, makes results difficult to compare. Further work will be needed to identify which immune mechanisms need to be triggered to confer complete, lasting protection, which antigens (or combination of) should be included in a potential vaccine, and the most appropriate delivery method.

### Live attenuated vaccines (LAVs)

LAVs circumvent a key issue presented by both inactivated and subunit or DNA preparations. Because they can successfully replicate within the host, they mimic natural infection thereby triggering both humoral and cellular pathways, and do not require adjuvants with co-stimulatory activity to enhance the magnitude and quality of the immune response. Additionally, some LAVs have been shown to elicit mucosal IgA antibodies, an important feature for vaccines administered via the oral route (oral immunization is a requirement for vaccines aimed at the wild boar population). That being said, LAVs pose a slight risk, as in rare cases attenuated strains may regain pathogenicity, causing the spread of disease, and they have the potential to cause post-vaccination reactions and side effects. Three main strategies have been employed in the generation of ASF LAVs, attenuation by cell passage, screening for naturally attenuated strains, and deletion of virulence-associated genes. To overcome some of the safety issues presented by LAVs, namely residual virulence, attempts have also been made at further deletion of virulence-associated genes in naturally attenuated strains, or adaptation to heterologous cell lines of gene deleted viruses. Depending on the immunogenicity of the deleted genes, these candidates may also be suitable for DIVA.

Attenuation by cell passage has its foundation in the observations of Manso and Sánchez that continuous passage of the circulating genotype I virulent ASFV strains in porcine bone marrow and kidney cells resulted in attenuation. Challenge experiments showed that pigs immunized with the attenuated strain were protected against the virulent strain, however, subsequent field trials were disastrous, with animals developing chronic ASF. Recent attempts using this strategy on genotype II strains currently circulating in Europe include attenuation of the Stavropol 01/08 strain by passage in the porcine lymphocyte hybrid cell line A4C2/9k and in the African green monkey kidney cell line CV-1 [44]. Although the resulting viruses lost pathogenicity, they failed to protect pigs against virulent challenge. Similarly, complete attenuation of an ASFV Georgia field isolate was achieved by passage in Vero cells, but infection with the attenuated strain did not confer protection [45]. The cell cultured adapted strain E75CV1, obtained from passage in the CV-1 cell line, conversely, was protective against the homologous E75 virus and demonstrated poor cross-protection against the heterologous BA71 [46].

Complementary to attenuation by cell passage is the screening of naturally attenuated non-hemadsorbing (non-HAD) strains and/or strains with reduced virulence which occur naturally during ASF epidemics. Examples include the non-HAD genotype I ASFV strains NH/P68 [18,47] and OURT88/3 [48] isolated in the Iberian Peninsula from chronically infected pigs and soft ticks, respectively, and which may have derived from the Manso cell passaged vaccine virus [49]; and the genotype II strain Lv17/WB/Rie1 isolated from wild boar in Latvia [17,50]. Pigs immunized with these strains were protected against challenge with homologous virulent strains [16–18,50], and in some cases, partial cross-protection against heterologous viruses was shown [14,47], with protection levels ranging from 66 to 100%. Though these naturally attenuated strains have the potential to be developed as LAVs, concern exists over their residual virulence, as a substantial proportion of the vaccinated pigs, at least at certain doses, develop unacceptable post-vaccination reactions.

A third strategy, critical to current ASF LAV research, is the rational deletion of virulence-associated genes or genes involved in the evasion of immune response by homologous recombination or CRISPR/Cas9 gene editing. This approach is being developed to improve the safety profile of naturally attenuated strains and for the attenuation of circulating virulent strains. Thus far several genes have been targeted: the hemagglutinin CD2v/EP402R, the thymidine kinase TK/K169R, the NF-κβ and NFAT inhibitor A238L, the apoptosis inhibitors A179L and A224L, the protein phosphatase-1 activator NL/DP71L, the genes involved in inhibiting the induction of Type I interferons such as MGFs 360 and 505 and the non-homologous I329L, K205R, DP148R and A276R, and genes implicated in the virulence of different ASFV virulent strains but whose mechanism of action is unclear, including 9GL/B119L, UK/DP96R, I177L, I226R, A137R, and E184L [8,51–53]. However, rational deletion does not always produce the intended result. For example, recent studies showed that deletion of the CD2v/EP402R gene (also known as 8DR) from the genotype I BA71 isolate attenuated the virus and that the resulting BA71ΔCD2 strain conferred protection against challenge with homologous and heterologous virulent viruses [54,55]. A similar deletion from the genotype II Georgia2010 isolate, ASFV-G-Δ8DR, however, did not significantly alter the virulence of the virus and produced clinical disease indistinguishable from that induced by the virulent parental strain [56]. Deletion of other specific virulence genes, such as TK/K169R [57,58], and NL/DP71L [59] has yielded similarly inconsistent outcomes, resulting in different phenotypes depending on the virus strain used. Depletion of specific virulence factors from naturally attenuated LAVs may likewise decrease their ability to protect against challenge with the parental virulent virus, as has been shown for OURT88/3 and NH/P68 [60,61]. These studies confirm that the results of ASFV genetic modifications may be unpredictable and that the effect of gene deletions on the ability of the virus to elicit immune protection is many times strain-specific. Also, concomitant deletion of virulence factors may, on occasion, yield weak viruses which cannot be grown *in vivo* or lack the ability to induce protective responses. This is the case with the experimental vaccine strains ASFV-G-Δ9GL/ΔCD2v [62], ASFV-G-Δ9GL/ΔMGF [63], and ASFV-GΔ9GL/ΔNL/ΔUK [64], all of which had significantly reduced protective potential compared to experimental strains lacking the individual ORFs. A notable exception is the Georgia2010 double mutant lacking 9GL and UK ASFV-G-Δ9GL/ΔUK which provided robust protection during challenge [65].

Another significant success with multiple gene deletions is the seven gene deleted attenuated strain HLj/18-7GD constructed by Chen et al. [66] which, according to the authors, was unable to revert to a virulent phenotype and provided complete long-term immunity (ten weeks) against a lethal ASFV challenge in pigs; safety of administration in pregnant sows was verified in this study, however only the absence of the targeted gene was assessed, hence, the involvement of off-target mutations cannot be ruled out. Efficacy against heterologous strains, safety after *per os* administration in wild boar, and absence of wild-type virus in tissues of vaccinated animals at longer times post challenge (more than one month) are other key factors that need to be evaluated and may determine this experimental vaccine’s effectiveness in real-world scenarios. Perhaps the most promising LAV candidate to date, however, is the ASFV-G-ΔI177L strain proposed by Borca et al. [67]; it can be administered by the intramuscular and oronasal route [68], inducing robust sterile immunity against challenge with the virulent parental ASFV Georgia isolate, involved in recent outbreaks, and proved effective in follow up field trials against the virulent Vietnamese strain TTKN/ASFV/DN/2019 [69]. Early this year, the Vietnamese Dabaco Group announced that testing of a commercial vaccine based on the ASFV-G-ΔI177L prototype was completed and they hoped to bring it to market in the second quarter of 2022, however, similar claims were made by Navetco National Veterinary JSC the previous year and failed to materialize [70]. Development of an ASFV-G-ΔI177L derivative strain, ASFV-G-ΔI177L/ΔLVR, that replicates efficiently in a stable porcine epithelial cell line and maintains the same level of attenuation, immunogenic characteristics, and protective efficacy in challenge studies [71], as well as real-time PCR assays to be used as genetic DIVA tests associated with the use of ASFV-G-ΔMGF, ASFV-G-Δ9GL/ΔUK, and ASFV-G-ΔI177L or cell culture adapted ASFV-G-ΔI177L/ΔLVR live attenuated vaccines in the field [72], are other recent advancements that make the development of a commercial ASF LAV vaccine in the near future a very likely occurrence. The ASFV-G-Δ9GL, ASFV-G-ΔMGF, ASFV-G-Δ9GL/ΔUK, ASFV-G-ΔI177L, and ASFV-G-ΔI177L/ΔLVR experimental vaccines were engineered and patented by the U.S. Department of Agriculture's (USDA) Agricultural Research Service (ARS).

Also of note are: the already mentioned BA71ΔCD2, which in addition to conferring solid protection against homologous and heterologous genotype I and II viruses presents the added advantage of being capable of stably growing in the commercial COS-1 cell line without needing previous adaptation; the genotype II naturally attenuated strain Lv17/WB/Rie1 which was the first to show solid protection in wild boar and also induces 100% protection in domestic pigs against virulent challenge with minimal and no clinical signs, respectively [73,74]; the genotype I naturally attenuated strain NH/P68 adapted to a cell line which has demonstrated a significant reduction of side effects and 100% protection against genotype II virulent virus [73]; and at earlier stages of development, but also showing effective immune responses in pigs, and providing complete protection against challenge with the parental strain, ASFV-SY18-ΔCD2v/ΔUK [75], SY18ΔI226R [76], ASFV-G-ΔA137R [77], and ASFV-G-ΔE184L [52], the first ASFV gene product experimentally shown to be a functional DIVA antigenic marker.

## Discussion

Veterinary vaccines are recognized as a cost-effective way to control infectious diseases, enhance the efficiency of food production, and prevent or reduce the transmission of zoonotic and foodborne infections to people. Efforts to design an effective vaccine against ASF, however, have been encumbered by several factors.

Strain diversity is a difficulty involved in the development of a vaccine against ASF. As already discussed, cross-protection is a multifactorial phenomenon that depends on numerous variables. Most experimental ASF vaccines target genotypes circulating in Europe and Asia with little attention paid to the 24 genotypes endemic in Africa, though, and they are unlikely to cross-protect against these more phylogenetically distant strains. Genetic variability amongst different breeds of pigs is another factor at play, reflected by the different patterns of pathogenesis and clinical outcomes observed across different regions of the world. The high level of genetic mutations that drive strain diversity in ASFV, coupled with the characterization of recombinant ASFV variants among Italian and South African isolates, and the identification of the MGF variable regions and other recombination hotspots, add to a growing body of evidence of the high genome plasticity of ASFV; detection of genotype II variants harbouring multiple natural mutations or deletions in recent disease outbreaks in China, the Dominican Republic and West Africa provides further evidence of this genome instability [78]. The ASF LAVs being developed are all based on modifications of ASFV genotype II and, to a lesser extent, genotype I strains, and reasonable concern has emerged over their use in non-endemic regions because of the potential for reversion to virulence. A potential already demonstrated by a six gene deleted experimental vaccine strain HLJ/18-6GD in the study by Chen et al. [66] that became more virulent during replication in pigs. Furthermore, there is a significant danger that because of the heavy socio-economic impact of ASF, stakeholders will be tempted to use apparently promising vaccine candidates before their effectiveness has been thoroughly evaluated. This may, in fact, already be happening. Over the past year, reports of genotype II gene deleted strains and genotype I strains highly similar to naturally attenuated European strains causing chronic disease in Chinese farms raised concerns about circulating unlicensed vaccines [79]. Although the number of know infections is low, if these hard-to-diagnose, low virulent strains were to spread widely, it could have severe consequences. Not the least of which is the potential for recombination events between the unlicensed strains and/or with future licensed vaccines, exacerbating concerns over vaccine safety and reversion to virulence.

Another safety issue is the risk of vaccine virus shedding which might occur under field conditions, with a proportion of naïve pigs becoming severely infected by exposure to large amounts of a live attenuated vaccine. This was recently demonstrated in a study by Lacasta et al. [46] comparing the course of *in vivo* infection caused by the two homologous ASFV strains E75 and the cell cultured adapted E75CV1, where two of the pigs inoculated with a lower dose of E75CV1 succumbed to challenge, an event which the authors speculated might have occurred because of direct in contact- transmission with pigs administered the high infectious-dose. Guidelines indicate that safety evaluation of LAVs should include long studies with many animals to assess the degree and stability of attenuation and assays be implemented that distinguish attenuated from both fully virulent and partially virulent strains to assess reversion. The production process should also be designed in such a way as to assess the stability of attenuation and for vaccines based on genetically modified organisms, an environmental risk assessment including the possibility of shedding of vaccine organisms following administration should also be included. This presents an added problem because the results of experiments with animal models do not always translate from the model animal to the pig [29,39] and experiments with pigs require strict biosafety level 3 (BSL3) biocontainment laboratories, tend to be extremely expensive, and are also environmentally and ethically difficult, considering the moderate to severe suffering associated with disease development, and the requirement that all animals be slaughtered at the end of the experiment, factors which hinder long-term monitoring of animals post-challenge [11]. Nevertheless, considering the results of studies such as Chen et al. and Lacasta et al., increasing biosafety tests for ASFV LAV prototypes should be mandatory in the future if they are to be commercialized.

In addition to being able to cross-protect, LAV formulations should also be compatible with wildlife immunization campaigns. Wild boar are highly susceptible to ASF and have been the major source of the spread of the disease in the current epizootic in Europe. In China, outbreaks in wild boar have also been reported, although there seems to be no direct connection with the infection in domestic pigs, as the causative strain is different from previously reported strains [80]. Still, given the large wild boar population, which is estimated to be in the millions, and its wide distribution, there is a high risk of endemicity, which would severely complicate control and eradication efforts. A specific vaccine for wild boar is thus a necessary investment. Unfortunately, most experimental LAVs have been administered by intramuscular injection and few studies on oral immunization have been carried out. To date, only the naturally attenuated strain Lv17/WB/Rie1 [17,74] has been shown to effectively protect wild boar against virulent challenge. However, only a small group of animals was used for the study, and evidence of vaccine virus shedding from orally vaccinated animals could mean that wild boar may be persistent carriers of the virus. A vaccine aimed at the wild boar population must be administered orally, thus a suitable delivery vehicle in the form of bait is needed, and it must also be sufficiently stable in the external environment to maintain potency when exposed to extreme environmental factors [51]. Following laboratory experiments, controlled field trials emulating free-ranging conditions should be performed before vaccination of natural populations is attempted.

Availability of an established, licensed porcine macrophage cell line and optimization of culture conditions for vaccine scale-up is another key constraint for ASFV vaccine development. With few exceptions [55,71], the reported LAV vaccine candidates rely on primary porcine macrophage and monocyte culture systems, and large-scale vaccine production using primary cells from animals is neither ethically acceptable nor feasible from a production perspective. Primary cells are laborious and costly to obtain and significant batch-to-batch variation occurs [11]. Further, there is evidence of genetic recombination between attenuated vaccine strains grown together in porcine alveolar macrophages (PAM)[81], which poses a risk of such events also occurring *in vivo* if or when different ASFV PAM vaccines start circulating. Although attempts have been made at the adaptation of some ASFV isolates to stable primate cell lines, such as VERO and CV-1, this process has generally resulted in genomic and phenotypic changes in the virus, sometimes negating protection [45]. Others have attempted to characterize porcine cell lines for susceptibility to ASFV infection and virus production capacity, but efficient production seems to be strain-dependent. Of note is the growth factor-dependent ZMAC-4 porcine macrophage cell line developed by Portugal et al. [82] which is susceptible to infection with eight different genotype I, II and VIII field isolates and supports high levels of replication of OURT88/3 without reducing its protective ability. In addition to a stable cell line, vaccine virus scale-up also requires high biocontainment for the production of the attenuated virus, ensuring vaccines are produced under high-quality standards and following the policy of the corresponding regulatory agencies.

It should be noted that even if all these challenges are surpassed, a vaccine against ASF would still be only one tool to control the disease. Since reporting of ASF outbreaks in disease-free regions results in trade restrictions and confirmation of freedom from disease is required to regain permission to export, a critical factor in the decision to vaccinate would be the disease status of the region and whether a diagnostic test was available to confirm freedom from disease. Thus, a DIVA strategy is essential. Two DIVA strategies for ASF LAVs have been discussed in the recent literature, one a multiplex real-time PCR targeting both the p72 gene of the wild-type ASFV strain and the deleted gene(s) of the LAV, the other the detection of antibodies induced by the deleted gene-encoded protein by enzyme-linked immunosorbent assay (ELISA) [8].

## Conclusion

Recent promising results with recombinant LAVs provide hope for a safe and effective vaccine against ASF. Coupled with specific, high-throughput DIVA diagnostic technologies and integrated into an ASF control and prevention policy based on a sound understanding of ASF epidemiology within the local socio-economic context, it may provide the basis for preventing, controlling, and eradicating ASF. It will be up to the scientific community, international agencies, governments, and industry to decide under which epidemiological scenarios they should be implemented considering the benefits provided by and disadvantages associated with the new vaccines as well as the different sensitivity for the ASF problem around the globe.
